# Association of Cardiac Rehabilitation with Improvement in High Sensitive C-Reactive Protein Post-Myocardial Infarction

**Published:** 2012-01-01

**Authors:** A Aminlari, M Jazayeri Shooshtari, A R Bakhshandeh

**Affiliations:** 1 Department of Physical Medicine and Rehabilitation, Shiraz University of Medical Sciences, Shiraz, Iran

**Keywords:** Cardiac Rehabilitation, C-reactive protein, Myocardial infarction

Dear Editor,

Atherosclerotic cardiovascular diseases are the major cause of death in middle-aged and older-adults.[1][2] Today, inflammation is regarded as the key pathogenic mechanism in both initiation and progression of atherosclerosis.[3]Inflammatory markers such as high sensitive C-reactive protein (CRP) have been used to identify patients at higher risk for coronary events. The prognostic value of CRP has been established for patients with acute coronary syndrome, stable coronary artery diseases[4]and in apparently healthy people.[5][6]

Comprehensive cardiac rehabilitation is probably the most effective approach for cardiovascular reduction and long-term care of cardiac patients as well as subjects with multiple coronary risk factors.[7][8] Secondary prevention, through cardiac rehabilitation program is now regarded as an essential component of contemporary management of patients with various presentations of coronary disease.[9]

There are some evidences that protective effects of cardiac rehabilitation may in part be related to lowering of inflammation. The goal of this prospective study was to assess the effect of cardiac rehabilitation on high sensitive CRP as a marker of increased risk of coronary artery disease.

The subjects were randomly selected from patients with recent anterior wall myocardial infarction referring to Alzahra Cardiac Rehabilitation Center in Shiraz from May to December 2007. Inclusion criteria were males and females aged 30-75 years with anterior wall MI in past 1 month. Patients were divided into two groups. Group A consisted of 30 participants who received cardiac rehabilitation and group B consisting of 26 participants which did not receive any cardiac rehabilitation program. CRP levels were assessed before starting the program and eight weeks later. We used CRP Quantitative Diagnostic Kit for serum and plasma-Photometry technique. The data were analyzed by Wilcoxon Signed Rank and Mann-Whitney tests.

The cardiac rehabilitation program included 8 weeks of exercise training, education and behavior modification therapy 3 times per week. The exercise training included arm and leg ergometry and treadmills. Behavioral modifications were smoking cessation, healthy nutrition, hypertension control, etc.

The average age of patients was 62.7 years old. At enrollment, 66% (n=20) of participants in cardiac rehabilitation (group A) had CRP levels in the intermediate and high-risk range of >1 mg/dl and after completion of cardiac rehabilitation only 27% (n= 5) of patients had elevated CRP levels (P=0.0009). In the group B, 73% (n=19) of participants had CRP levels >1 mg/dl and after eight weeks, 53% (n=14) of them had CRP levels >1 mg/dl (p=0.50).

A higher CRP level was observed in patients <65 years of age participating in cardiac rehabilitation compared to those >65 years, but both age groups demonstrated significant improvements in CRP postcardiac rehabilitation. This study showed a significant decrease in CRP levels in patients with cardiac disease after participation in cardiac rehabilitation program. The findings showed that cardiac rehabilitation decreased vascular inflammation accompanying improvement in risk factors.

Regardless of gender, there was a significant decrease in CPR at the completion of cardiac rehabilitation. The decrease in inflammation along with previously described benefits of cardiac rehabilitation supports the recommendations of American Heart Association and American Association of Cardiovascular and Pulmonary Rehabilitation that participation in cardiac rehabilitation should be a part of comprehensive care of cardiac patients.

The findings in our study had two important clinical implications. First, the potential independent effect of cardiac rehabilitation in lowering CRP levels was demonstrated which is independent from weight in our study. The non-pharmacologic mechanism for this observation may be due to dietary modifications, weight management and exercise components of the program. Secondly, the decrease in vascular inflammation associated with cardiac rehabilitation is evident regardless of gender, age and metabolic syndrome.

## References

[1] (1993). Rehabilitation after cardiovascular diseases, with special emphasis on developing countries: report of WHO committee. World Health Organ Tech Rep Ser.

[2] Ades PA (2001). Cardiac rehabilitation and secondary prevention of coronary heart disease. N Engl J Med.

[3] Pearson TA, Mensah GA, Alexander RW, Anderson JL, Cannon RO 3rd, Criqui M, Fadl YY, Fortmann SP, Hong Y, Myers GL, Rifai N, Smith SC Jr, Taubert K, Tracy RP, Vinicor F (2003). Centers for Disease Control and Prevention; American Heart Association. Markers of inflammation and cardiovascular disease: Application to clinical and public health practice: A statement for healthcare professionals from the center for Disease control and prevention and the American Heart Association. Circulation.

[4] Zebrack JS, Anderson JL, Maycock CA, Horne BD, Bair TL, Muhlestein JB (2002). Intermountain Heart Collaborative (IHC) Study Group. Usefulness of high-sensitivity C-reactive protein in predicting long-term risk of death or acute myocardial infarction in patients with unstable or stable angina pectoris or acute myocardial infarction. Am J Cardiol.

[5] Ridker PM, Cushman M, Stampfer MJ, Tracy RP, Hennekens CH (1997). In­flammation, aspirin and the risk of cardiovascular disease in apparently healthy men.. N Engl J Med.

[6] Esbah O, Gursoy G, Kirnap NG, Cetiner H, Demirbas B, Acar Y, Bayram M (2011). Relation of resistin levels with C-reactve protein, homocysteine and uric acid in smokers and non-smokers. J Res Med Sci.

[7] Fletcher GF, Balady G, Blair SN, Blumenthal J, Caspersen C, Chaitman B, Epstein S, Sivarajan Froelicher ES, Froelicher VF, Pina IL, Pollock ML (1999). Statement on exercise: benefits and recommendations for physical activity programs for all Americans. A statement for health professionals by the Committee on Exercise and Cardiac Rehabilitation of the Council on Clinical Cardiology, American Heart Association.. Circula­tion.

[8] Balady GJ, Ades PA, Comoss P, Limacher M, Pina IL, Southard D, Williams MA, Bazzarre T (2000). Core com­ponents of cardiac rehabilita­tion/secondary prevention programs: A statement for healthcare profes­sionals from the American Heart As­sociation and the American Associa­tion of Cardiovascular and Pulmo­nary Rehabilitation Writing Group.. Circulation.

[9] O'Connor GT, Buring JE, Yusuf S, Goldhaber SZ, Olmstead EM, Paffenbarger RS Jr, Hennekens CH (1989). An overview of randomized trials of re­habilitation with exercise after myocardial infarction.. Circulation.

